# Impact of social determinants of health on physical therapy utilization in home health and outpatient settings: a retrospective cohort study

**DOI:** 10.1093/ptj/pzag090

**Published:** 2026-08-29

**Authors:** Michael Markee, Hyunkyu Ko, Brook I Martin, Amit Kumar

**Affiliations:** Department of Physical Therapy and Athletic Training, Saint Louis University, St. Louis, MO 63104, United States; Department of Orthopaedics, Orthopaedic Center, University of Utah, Salt Lake City, UT 84108, United States; Department of Orthopaedics, Orthopaedic Center, University of Utah, Salt Lake City, UT 84108, United States; Department of Physical Therapy and Athletic Training, University of Utah, Salt Lake City, UT 84108, United States; Center for Research on Race Health Justice and Public Policy, University of Utah, Salt Lake City, UT 84112, United States; Department of Population Health Sciences, University of Utah, Salt Lake City, UT 84112, United States

**Keywords:** Social determinants of health, lower extremity joint replacement, physical therapy, social vulnerability, Medicare

## Abstract

**Importance:**

Home health and outpatient physical therapy are important for recovery after lower extremity joint replacement (LEJR); yet, their use may be influenced by social determinants of health (SDOH).

**Objective:**

The objective of this study was to identify SDOH factors at the patient-, hospital-, and regional-levels that are associated with the use of home health and outpatient physical therapy after LEJR.

**Design:**

This cross-sectional study used 100% Medicare fee-for-service claims. Hierarchical regression models assessed associations of patient-, hospital-, and regional-level SDOH with home health and outpatient physical therapy utilization.

**Setting:**

Not applicable.

**Participants:**

Medicare beneficiaries who were 66 years old and older in 2018 and who were eligible for participation in the Comprehensive Care for Joint Replacement (CJR) program.

**Exposure(s):**

SDOH at the patient level (race, dual eligibility status), hospital level (percentage of Disproportionate Share Hospital patients), and regional level (themes from the Social Vulnerability Index).

**Main Outcome and Measure(s):**

Number of physical therapy visits in home health and outpatient settings within 90 days after discharge following an LEJR surgery, stratified by hip replacement and knee replacement.

**Results:**

This study included 74,937 patients undergoing joint replacement from January 1 to September 30, 2018. At the patient level, Black patients had more home health and outpatient physical therapy visits than White patients following both knee and hip replacement. Dual-eligibility status was associated with more home health visits but fewer outpatient physical therapy visits. At the regional level, greater socioeconomic vulnerability was associated with more home health visits and outpatient physical therapy visits. Greater vulnerability in housing type and transportation was associated with more home health visits but fewer outpatient physical therapy visits.

**Conclusions:**

SDOH were associated with greater home health and outpatient physical therapy utilization following hospital discharge after joint replacement.

**Relevance:**

Incorporating SDOH into discharge planning may promote equitable access to community-based rehabilitation following LEJR.

## Introduction

Lower extremity joint replacement (LEJR) procedures (total hip and total knee) are the most common procedures among Medicare beneficiaries,^1^ with the number of surgeries in the United States expected to grow to over 4 million by 2040.^2^ Despite the growth in LEJR volume, the cost for the surgery and postacute care has decreased in both hip and knee groups, particularly after the inception of bundled payment programs.^3^^,^^4^ This has resulted in shifting postacute rehabilitation care from institution-based to home settings.^5^^,^^6^ Physical therapy serves as an important component of this postacute care in restoring the patients’ functional status following total knee replacement^7–12^ and total hip replacement.^13^^,^^14^ Research shows that patients who have undergone knee replacement generally achieve better functional and overall health outcomes when they receive physical therapy, whether in acute, outpatient, or home health settings.^15^^,^^16^

Disparities in the receipt of LEJR and the subsequent postacute care exist at both individual and regional levels and are strongly influenced by social determinants of health (SDOH).^17^ Counterintuitively, individual-level and region-level SDOH function at different stages of a patient’s care journey. Individual factors, such as race, income, and education, directly influence patient choices and patient outcomes. In contrast, regional factors, including neighborhood-level determinants or deprivation shape the broader structural context that determines access to and availability of care. Although both levels play distinct roles across the patient care continuum, there is limited research examining how individual-level and region-level SDOH influence postacute rehabilitation care following LEJR, representing an important gap. Research indicates that Black older adults receive fewer LEJRs,^18^^,^^19^ receive more home health physical therapy (following knee replacement),^20^^,^^21^ and are less likely to receive outpatient physical therapy after LEJR.^22^ Moreover, individuals residing in socially vulnerable communities receive fewer LEJRs,^23^ are less likely to receive outpatient physical therapy within 90 days of surgery,^22^ and are more likely to return to the emergency department within 90 days of joint replacement.^24^^,^^25^ Previous research utilized the Distressed Communities Index to measure economic distress and found that patients with knee replacements living in more distressed communities exhibited higher rates of hospital readmission and a greater likelihood of receiving postoperative medication prescriptions.^26^ Following LEJR, older adults often require community-based physical therapy, which may be influenced by SDOH. However, to our knowledge, no research has examined how patient and regional SDOH, including social vulnerability, affect community-based physical therapy use after LEJR.

Starting in 2016, under the mandatory Comprehensive Care for Joint Replacement (CJR) program, hospitals were reimbursed for services provided to traditional Medicare beneficiaries related to LEJR for 90 days following discharge from the acute-care hospital stay (index hospitalization), holding hospitals financially accountable for the quality and cost of care during that period.^1^ A prominent point of savings in CJR model was postacute care, with fewer patients discharged to institution-based postacute care settings, such as skilled nursing facilities and inpatient rehabilitation facilities, and more patients discharged to the community.^3^^,^^4^ But CJR model fell short in addressing patient-level^27–30^ and region-level SDOH,^31^ leading to disparities in LEJR procedures and postacute care. As research continues to examine how SDOH at various levels affect care delivered to patients, there remains a notable gap in our understanding of how social risk factors at the patient, hospital, and regional levels affect the utilization of physical therapist services in home health and outpatient settings following LEJR. Addressing this gap is essential for enhancing the delivery of community-based physical therapist services (home health and outpatient) and health outcomes. The objective of this study is to examine patient-, hospital-, and regional-level SDOH factors associated with home health and outpatient physical therapy utilization following acute care hospital discharge after LEJR.

## Methods

### Study design

We conducted a retrospective analysis of traditional Medicare beneficiaries who were 66 years old and older and who underwent LEJR procedures, including knee and hip replacements. We used 100% of Medicare claims data accessed through the Virtual Research Data Center.^32^ This analysis focused on Medicare fee-for-service beneficiaries who had undergone LEJR and were discharged from CJR-participating acute care hospitals (*n* = 493) or non–CJR-participating hospitals but eligible for CJR (*n* = 475). The study period for LEJR procedures was between January 1, 2018, and September 30, 2018, and for physical therapy utilization was 90 days following surgery between January 1, 2018, and December 31, 2018. To minimize potential confounding factors, we included patient-, hospital-, and regional-level covariates in our model.

### Data and outcomes

We identified Medicare beneficiaries who were admitted for an inpatient LEJR procedure defined by Medicare Severity Diagnosis Related Groups (MS-DRG) codes 469 and 470 by using a 100% sample of Medicare Part A and Part B fee-for-service claims (MS-DRG code definitions are shown in Suppl. Table 1). We excluded beneficiaries who were eligible for Medicare through end-stage renal disease or Social Security Disability Insurance or who were enrolled in a Medicare Advantage plan. LEJRs in Medicare Advantage plans were not part of the CJR program and are reimbursed differently than those in traditional Medicare, potentially changing the incentive for postacute care services. Hospitals already participating in the voluntary Bundled Payment for Care Improvement for LEJR were excluded because they were not eligible for CJR. To ensure that each beneficiary is represented only once in the analysis, we restricted our sample data to their first occurrence of LEJR during the study period.

#### Primary outcomes

The study outcome was the number of home health and outpatient physical therapy visits from January 1, 2018, through December 31, 2018. Physical therapy visits were identified as those with a physical therapy-specific revenue center code in home health claims and outpatient claims data (Suppl. Table 1). Home health physical therapy visits were defined as the total number of days within a 90-day episode of care when at least 1 home health physical therapy claim was submitted. Similarly, outpatient physical therapy visits were defined as the total number of days within a 90-day episode of care when at least 1 outpatient physical therapy claim was submitted.

#### Covariates

We included patient-level factors such as age, race, sex, Charlson Comorbidity Index prior to surgery, MS-DRG codes, discharge status, and Medicare–Medicaid dual eligibility (dual-eligible) status. We also included hospital-level factors such as CJR status, teaching status, for-profit status, percentage of patients who had dual eligibility (Disproportionate Share Hospital patient percentage), number of beds, hospital census as a percentage of capacity, and LEJR volume. Hospitals with a high percentage of patients with dual eligibility were included, serving as a strong proxy for social risk and correlating with greater patient complexity, increased resource utilization, and poorer outcomes.^33^ Hospital-level data and CJR participation were acquired from the Centers for Medicare & Medicaid Services Impact Files and the CJR website, respectively.^1^^,^^34^

To capture the SDOH at the regional level, we obtained data on the Social Vulnerability Index (SVI) from the Centers for Disease Control and Prevention.^35^ Designed in 2011 to address hazards and public health emergencies,^35^ the ZIP Code–level SVI measure quantifies the vulnerability of communities on the basis of indexes for socioeconomic status, household characteristics (household members who are elderly or disabled, children, single parents), minority status, and housing type and transportation (nontraditional housing structure, crowding, no vehicle). These indexes range from 0 to 1, with 1 representing the most vulnerable in each category. The SVI has been used to study region-level SDOH^23^^,^^36–38^ but has yet to be applied to a population of Medicare patients with joint replacement.

### Data analysis

To examine the SDOH variables associated with home health and outpatient physical therapy visits, we employed both univariate and multivariate analyses, stratifying patients into groups on the basis of whether they underwent hip or knee replacement. Since we have right-skewed continuous outcome variables, a generalized linear model with gamma distribution and log-link function was used to estimate the effect of the covariates. However, we presented the ordinary least squares results because they yielded comparable results to the generalized linear model and are more straightforward to interpret. Inferences did not differ between the 2 model specifications. The results from the generalized linear model analysis with gamma distribution can be found in Supplementary Tables 2 and 3.

In our main analysis, we conducted several regression analyses to explore how patient, hospital, and region-level SDOH factors influence home health and outpatient physical therapy utilization in patients discharged to home from the index hospitalization. The 3 models of analyses included unadjusted, adjusted with patient-level variables, and adjusted with patient-, hospital-, and region-level variables. A variance inflation factor was used to assess collinearity among the covariates used in the regression models (Suppl. Table 4). Secondary sensitivity analyses included patients discharged from acute care hospitals to institutional postacute care settings, as those transitioning to home after institutional postacute settings may have different community-based physical therapy needs than patients discharged directly home. All regressions included stratum-specific fixed effects because of the probability of being selected as a CJR or a non-CJR hospital across strata. We used robust SEs. All findings are presented with 2-tailed tests, and statistical significance is determined at a *P* value threshold of <.05. This study was exempted from institutional review board of the University of Utah. Stata (version 18.0-MP; StataCorp LLC; College Station, TX, USA) was used for all analyses.

### Role of the funding source

The funders played no role in the design, conduct, or reporting of this study.

## Results

### Sample characteristics

The final analysis included 74,937 LEJR episodes. Of these, 35,657 came from hospitals participating in the CJR program, with 59.1% being knee replacements and 40.9% being hip replacements. The remaining 39,280 episodes were from non-CJR hospitals, including 58.5% knee and 41.5% hip replacements. (Suppl. Figure 1). Table 1 presents the characteristics of patients grouped by tertiles (low, medium, and high) of the overall SVI to ensure balanced group sizes. Across hip and knee groups, the highest SVI tertile had a lower proportion of White patients, a higher proportion of dual-eligible patients, and higher Charlson Comorbidity Index scores. These patients were also more likely to be discharged from hospitals serving a higher proportion of dual-eligible patients.

**Table 1:** Descriptive characteristics of patients undergoing hip and knee replacement procedures in CJR-eligible hospitals, stratified by overall SVI categories*^a^*

Variables	Hip (*n* = 30,868)				Knee (*n* = 44,069)			
	Low SVI*^a^* (*n* = 10,482; 34.0%)	Medium SVI*^a^* (*n* = 10,074; 32.6%)	High SVI*^a^* (*n* = 10,312; 33.4%)	*P* *^b^*	Low SVI*^a^* (*n* = 14,612; 33.2%)	Medium SVI*^a^* (*n* = 14,812; 33.6%)	High SVI*^a^* (*n* = 14,645; 33.2%)	*P* *^b^*
Patient-level characteristics								
Age, y, mean	74.7	74.5	74.6	.170	74.1	74.1	74.1	.880
White, %	92.5	91.1	90.0	.001*^b^*	89.5	88.6	85.5	<.001*^b^*
Female, %	63.8	62.5	62.6	.103	66.1	67.1	66.3	.142
Dual eligible, %	4.8	5.5	7.3	<.001*^b^*	7.2	8.0	12.2	<.001*^b^*
Average Charlson Comorbidity Index 365 d prior to surgery	1.71	1.72	1.82	<.001*^b^*	1.80	1.79	1.94	<.001*^b^*
MS-DRG 469, %	2.3	2.9	3.2	<.001*^b^*	2.7	3.2	3.1	.025*^b^*
MS-DRG 470, %	97.7	97.1	96.8	<.001*^b^*	97.3	96.8	96.9	.025*^b^*
Discharge destination, %								
Routine discharge	5.8	6.2	6.7	.031*^b^*	6.5	7.3	7.9	<.001*^b^*
Discharge to SNF	18.9	16.3	17.6	<.001*^b^*	20.2	16.9	20.0	<.001*^b^*
Discharge to HHS	71.6	72.6	68.9	<.001*^b^*	69.9	71.5	66.0	<.001*^b^*
Discharge to IRF	2.9	4.0	5.8	<.001*^b^*	2.7	3.5	5.1	<.001*^b^*
Hospital-level characteristics								
Teaching status, %	47.8	61.2	58.7	<.001*^b^*	47.9	58.1	55.5	<.001*^b^*
For-profit status, %	13.2	16.9	22.3	<.001*^b^*	15.1	20.4	27.1	<.001*^b^*
DPP, %	20.9	22.0	27.1	<.001*^b^*	20.7	22.1	28.3	<.001*^b^*
No. of hospital beds	372	380	369	.040*^b^*	355	351	354	.507
Hospital census as percentage of capacity, %	58.8	59.1	59.8	<.001*^b^*	58.5	57.3	58.5	<.001*^b^*
LEJR volume as percentage of census, %	14.2	18.6	15.7	<.001*^b^*	15.6	20.1	16.7	<.001*^b^*
Region-level characteristics obtained through SVI								
Socioeconomic status index	0.14	0.40	0.76	<.001*^b^*	0.14	0.40	0.76	<.001*^b^*
Household characteristics index	0.25	0.34	0.63	<.001*^b^*	0.24	0.35	0.66	<.001*^b^*
Racial and ethnic minority status index	0.31	0.47	0.67	<.001*^b^*	0.31	0.46	0.67	<.001*^b^*
Housing type and transportation index	0.24	0.59	0.77	<.001*^b^*	0.23	0.58	0.75	<.001*^b^*
Overall Social Vulnerability Index	0.13	0.43	0.79	<.001*^b^*	0.12	0.43	0.79	<.001*^b^*
Mean no. of home health and outpatient visits								
Home health	8.2	8.3	9.0	<.001*^b^*	8.6	9.0	9.5	<.001*^b^*
Outpatient	2.2	2.0	1.7	<.001*^b^*	3.7	4.0	3.5	<.001*^b^*

a SVI was categorized into tertiles (low, medium, and high) on the basis of the distribution in our analytic cohort to ensure balanced group sizes.

b *P* values of <.05.

c CJR = Comprehensive Care for Joint Replacement; DPP = Disproportionate Share Hospital patient percentage; HHS = home health services; IRF = inpatient rehabilitation facility; LEJR = lower extremity joint replacement; MS-DRG = Medicare Severity Diagnosis Related Group; SNF = skilled nursing facility; SVI = Social Vulnerability Index.

### Effect of SDOH risk factors on home health physical therapy

In the multivariate analysis for home health physical therapy, patients who identified as Black or who had dual eligibility received more visits in both hip and knee groups (Table 2). Patients from hospitals with a high percentage of patients with dual eligibility received more visits in both groups, as well. As the percentage of patients with dual eligibility who were served by the hospital increased by 1 point, the number of home health visits increased by 0.62 in the hip group and by 1.06 in the knee group. At the regional level, patients from communities with more vulnerability in socioeconomic status, or housing type and transportation received more home health physical therapy visits in both groups. While a 1-percentage-point change in the SVI is the unit of measure in our regression analysis, larger variations of the index may provide more meaning in interpreting the data. As the socioeconomic status SVI increased by 10 percentage points, the number of home health visits increased by 9.7 in the hip group and 4 in the knee group. As the housing type and transportation SVI increased by 10 percentage points, the number of home health visits increased by 3.3 and 8.4 in the hip and knee groups, respectively. However, as the race and ethnicity SVI increased by 10 percentage points, the number of home health visits decreased by 3.6 in the hip group. The Supplementary Materials show the complete results of the multilevel analyses of home health visits when using patient-level only as well as patient-, hospital-, and regional-level variables (Suppl. Tables 5–9).

**Table 2:** Association between social determinants of health and home health physical therapy visits for patients discharged to home*^a^*

Variables	Hip replacement		Knee replacement	
	Estimated coefficient (95% CI)	*P* *^b^*	Estimated coefficient (95% CI)	*P* *^b^*
Race (reference: White)				
Black	0.53 (0.25 to 0.81)	<.001*^b^*	0.37 (0.14 to 0.60)	.001*^b^*
Other	−0.09 (−0.32 to 0.15)	.471	0.42 (0.22 to 0.61)	<.001*^b^*
Dual eligibility (reference: non-dual eligible)				
Dual eligible	1.29 (0.98 to 1.61)	<.001*^b^*	0.76 (0.54 to 0.97)	<.001*^b^*
Disproportionate share hospital patient percentage	0.62 (0.10 to 1.13)	.019*^b^*	1.06 (0.64 to 1.47)	<.001*^b^*
Social Vulnerability Index themes				
Socioeconomic status index	0.97 (0.71 to 1.23)	<.001*^b^*	0.40 (0.18 to 0.61)	<.001*^b^*
Household characteristics index	−0.03 (−0.25 to 0.19)	.797	0.07 (−0.11 to 0.25)	.471
Race and ethnicity index	−0.36 (−0.66 to −0.05)	.022*^b^*	−0.02 (−0.27 to 0.23)	.873
Housing type and transportation index	0.33 (0.12 to 0.54)	.002*^b^*	0.84 (0.67 to 1.02)	<.001*^b^*

^a^ Multivariate analyses of home health physical therapy visits with covariates related to social determinants of health for patients discharged to home.

^b^ *P* values of <.05.

### Effect of SDOH risk factors on outpatient physical therapy

In the multivariate analysis for outpatient physical therapy, patients who identified as Black received more physical therapy visits than patients who identified as White, but patients with dual eligibility had fewer visits in both hip and knee groups (Table 3). Patients discharged from hospitals with a high percentage of patients with dual eligibility received fewer visits in both groups. As the percentage of patients with dual eligibility who were served by the hospital increased by 1 point, the number of outpatient visits decreased by approximately 3 visits in the hip and knee groups. At the regional level, patients from communities with more vulnerability in socioeconomic status received more visits in the knee group, while vulnerability in housing and transportation resulted in fewer visits in both groups. A 10-percentage-point increase in the socioeconomic status SVI equated to a 7.2 outpatient visit increase in the knee group. Conversely, as the housing type and transportation SVI increased by 10 percentage points, the number of outpatient visits decreased by 7 and 6.6 in the hip and knee groups, respectively. The Supplementary Materials show the complete results of the multilevel analyses of outpatient visits when using patient-level only, and patient-, hospital-, and regional-level variables (Suppl. Tables 10–14).

**Table 3:** Association between social determinants of health and outpatient physical therapy visits for patients discharged to home*^a^*

Variables	Hip replacement		Knee replacement	
	Estimated coefficient (95% CI)	*P* *^b^*	Estimated coefficient (95% CI)	*P* *^b^*
Race (reference: White)				
Black	0.92 (0.20 to 1.64)	.012*^b^*	0.56 (0.03 to 1.09)	.040*^b^*
Other	0.78 (0.07 to 1.50)	.032*^b^*	0.33 (−0.16 to 0.81)	.189
Dual eligibility (reference: non-dual eligible)				
Dual eligible	−0.86 (−1.63 to −0.09)	.028*^b^*	−2.04 (−2.50 to −1.57)	<.001*^b^*
Disproportionate share hospital patient percentage	−3.14 (−4.67 to −1.61)	<.001*^b^*	−2.94 (−3.99 to −1.88)	<.001*^b^*
Social Vulnerability Index themes				
Socioeconomic status	−0.20 (−0.96 to 0.56)	.603	0.72 (0.14 to 1.30)	.014*^b^*
Household characteristics	0.26 (−0.33 to 0.86)	.380	0.41 (−0.04 to 0.85)	.076
Race and ethnicity	0.36 (−0.52 to 1.23)	.425	−0.24 (−0.89 to 0.41)	.470
Housing type and transportation	−0.70 (−1.29 to −0.11)	.021*^b^*	−0.66 (−1.11 to −0.20)	.005*^b^*

^a^ Multivariate analyses of outpatient physical therapy visits with covariates related to social determinants of health for patients discharged to home.

^b^ *P* values of <.05.

## Discussion

This is the first study to our knowledge to identify patient-, hospital-, and regional-level SDOH factors contributing to differences in physical therapy utilization following LEJR. Our findings indicate that patients with dual eligibility (indicating lower socioeconomic status at the patient level) used more home health physical therapy and less outpatient physical therapy after both hip and knee replacements. Similarly, patients discharged from hospitals with a higher percentage of patients with dual eligibility received more home health physical therapy visits and fewer outpatient physical therapy visits. Likewise, at the regional level, patients living in communities with more multiunit or mobile homes, crowded housing, or a lack of transportation also received more home health and fewer outpatient physical therapy visits. These results suggest patients who are more at risk in socioeconomic status, housing, and transportation are likely to receive more home health and less outpatient physical therapy following LEJR.

Patients with dual eligibility may receive different levels of care intensity than others. When analyzing post-acute care patterns of patients 45 years and older discharged to home across 4 states, Medicaid beneficiaries were less likely to receive home health physical therapy following total knee replacement than Medicare or privately insured patients.^39^ Our finding of more home health visits in this group adds more specificity with the use of a national sample of age-based Medicare beneficiaries. With state-to-state differences in individuals with dual eligibility,^40^ our population of individuals with dual eligibility is more representative of the Medicaid population. Regarding outpatient visits, our finding that patients with dual eligibility received less outpatient care aligns with prior work showing that patients with dual eligibility have fewer outpatient visits and subspecialty visits.^41^ Patients with dual eligibility may not have the resources to attend outpatient physical therapy or may not understand the details of their Medicaid coverage which reimburses outpatient physical therapy and varies from state to state.^42^ This information is significant, as patients with dual eligibility have worse outcomes following LEJR than those who are eligible for Medicare alone.^43^ For those who receive home health followed by outpatient care, these findings suggest that progress achieved in the home health setting may not be sustained due to a lack of follow-up care in the outpatient setting.

The largest coefficients were seen in the hospitals serving more patients with dual eligibility, commonly referred to as safety net hospitals.^44^ Similar to dual eligibility at the patient level, we found that as the percentage of patients with dual eligibility in the hospital increased, patients received more home health visits and fewer outpatient visits. Patients with dual eligibility are typically low-income, high-risk individuals, and hospitals serving higher proportions of these patients may have limited resources to support postacute care continuity and access to nearby community-based physical therapist services. These factors create compounding challenges in care delivery and outcomes. Patients from safety net hospitals are more likely to rank their patient experiences lower,^45^ and are more likely to be discharged to an institution rather than home,^46^ particularly when the hospital is participating in an LEJR alternative payment model.^47^ It is possible that, with more patients discharged to institutional settings, safety net hospitals lack the resources to provide options for community-based care. Furthermore, options for outpatient rehabilitation may be limited surrounding these hospitals. Policies that address the cost and quality of postacute care for LEJR should include resources to facilitate outpatient rehabilitative care for patients discharged from safety net hospitals. Future research should investigate the disparities in resources available during the discharge planning process at these hospitals, as well as the availability of outpatient physical therapy facilities in proximity to these hospitals.

Our findings related to SVI themes provide insight into these patterns and further expand the literature in this domain. The theme with the most consistent findings was with those living in vulnerable communities characterized by unstable housing situations, such as multiunit or mobile homes or crowded homes, and lacking access to transportation. This complements other studies that show patients who are from more vulnerable areas are less likely to receive LEJR^23^ and are more likely to be discharged to an institution.^38^ Specific to physical therapy, a lack of transportation has been associated with fewer outpatient visits.^48^ While SVI and physical therapy utilization has been studied in patients with a stroke,^49^ ours is the first known study to examine the relationship of SVI in community-based physical therapy utilization in an orthopedic population. Providers who serve patients in these more vulnerable areas should encourage home health physical therapy or seek resources if the patients are more appropriate for outpatient care.

Our results regarding other patient-level SDOH variables build upon findings from other studies. Patients who are Black are less likely to receive LEJR^50^ and recent research has shown the disparity is not improving.^51^^,^^52^ Non-White patients and those with a lower household income receive more home health physical therapy visits^20^^,^^21^ and are less likely to receive outpatient physical therapy after receiving total knee replacement.^22^ This partially supports our patient-level variable of race, with patients who are Black receiving more home health and more outpatient physical therapy visits compared to their White counterparts. However, at the regional level, the only association between the race and ethnicity SVI and physical therapy utilization was in fewer home health visits for patients with hip replacements in communities with more minorities. The lack of agreement between race at the patient and regional levels speaks to the different levels at which this variable is measured. While race at the individual level was significant in most settings, the associated SVI theme measured at the regional level was not as impactful.

Home health and outpatient care should not be viewed as equal in nature, as not all patients receive physical therapy in both settings. Research indicates patients receiving only outpatient physical therapy may have better outcomes compared to those receiving both home health and outpatient physical therapy following joint replacement.^53^ Moreover, it is possible that SDOH variables may have a different impact on home health physical therapy than on outpatient physical therapy. For instance, a negative finding in a variable pertaining to transportation may be more consequential for services in the outpatient than in the home setting since a patient needs transportation for one setting but not the other. Nonetheless, the clinical meaningfulness of our results must consider effect sizes. A change of 1 visit or less in the context of a patient’s entire plan of care may have little effect on the clinical outcomes. Further research should correlate these findings for patients in home health and/or outpatient physical therapy with patient-centered outcomes.

### Limitations

There are several limitations with this analysis. First, any generalization of the multivariate analyses should be analyzed with caution. Using a population of Medicare beneficiaries from CJR-eligible hospitals, this program was designed for hospitals within metropolitan statistical areas. These findings may not be generalizable to micropolitan or rural areas. However, research shows most total knee replacements are performed in metropolitan areas.^54^ Second, while our dataset used Medicare, the largest insurer for patients over 65 years of age, LEJRs are increasingly performed in younger patients, as well.^2^^,^^55^ Younger patients may have different responses to SDOH at the individual or regional level. Third, as this is a secondary data analysis of claims, there is no information on the specific treatment provided or the patient’s functional or pain status. While the Outcome Assessment Information Set can be used for Medicare beneficiaries in home health, there is no such functional outcome measure reported in the outpatient setting. Fourth, we included only patients enrolled in the fee-for-service plan, and our results may not apply to patients enrolled in Medicare Advantage plans. Lastly, this cross-sectional analysis cannot determine if there was a change over time in these SDOH variables in response to participation in CJR.

## Conclusions

Patients from lower socioeconomic backgrounds, discharged from safety net hospitals, or living in underserved communities were more likely to receive home health physical therapy and have fewer outpatient physical therapy visits after LEJRs. Our results expand past research by exploring the individual, hospital, and regional SDOH factors contributing to disparities in community-based physical therapy utilization. With the rise of episode-based payment models for LEJRs, CMS should incentivize providers to address SDOH factors in discharge planning and enhance community-based rehabilitation. Future research should compare outcomes in those receiving home health and outpatient physical therapy, with SDOH as mediating factors, to determine if there is an optimal range of community-based physical therapy visits. The SVI offers more granular data to providers on what SDOH may contribute to improve postacute rehabilitation care continuity.

## Supplementary Material

PTJ-2025-0855_R1_SupplementalFiles_SDOHinPT_editorialofficeversion_pzag090

## Data Availability

Data utilized in this study are available through the Centers for Medicare and Medicaid Services. As these data include protected health information, data are not available for sharing.
