# Supplementary material for: Impact of social determinants of health on physical therapy utilization in home health and outpatient settings: a retrospective cohort study
Source: Phys Ther. 2026 Aug 29;106(10):pzag090. doi: 10.1093/ptj/pzag090 (PMC13647087; doi:10.1093/ptj/pzag090)

Supplemental Figures and Tables

TABLE 1: Physical therapy-specific revenue center codes and diagnosis related group (DRG) codes used in claims data

TABLE 2: Association between social determinants of health and home health physical therapy visits for patients discharged to home using generalized linear model analysis with a gamma distribution.

TABLE 3: Association between social determinants of health and outpatient physical therapy visits for patients discharged to home using generalized linear model analysis with a gamma distribution.

TABLE 4: Variance Inflation Factor for covariates included in the full regression models.

TABLE 5: Multivariate analyses of home health physical therapy visits with covariates related to social determinants of health for patients regardless of discharge destination.

TABLE 6: Multivariate analyses of home health physical therapy visits with patient level covariates for patients discharged home.

TABLE 7: Multivariate analyses of home health physical therapy visits with patient level covariates for patients regardless of discharge destination

TABLE 8: Multivariate analyses of home health physical therapy visits with patient, hospital, and regional level characteristics for patients discharged home.

TABLE 9: Multivariate analyses of home health physical therapy visits with patient, hospital, and regional level characteristics for patients regardless of discharge destination.

TABLE 10: Multivariate analyses of outpatient physical therapy visits with covariates related to social determinants of health for patients regardless of discharge destination.

TABLE 11: Multivariate analyses of outpatient physical therapy visits with patient level covariates for patients discharged home.

TABLE 12: Multivariate analyses of outpatient physical therapy visits with patient level covariates for patients regardless of discharge destination

TABLE 13: Multivariate analyses of outpatient physical therapy visits with patient, hospital, and regional level characteristics for patients discharged home.

TABLE 14: Multivariate analyses of outpatient physical therapy visits with patient, hospital, and regional level characteristics for patients regardless of discharge destination.

FIGURE (Supplement). Exclusion criteria flow diagram

34

TABLE 1 (Supplement): Physical therapy-specific revenue center codes and diagnosis related group (DRG) codes used in claims data

	Code	Description
Revenue center codes to identify PT services	0420	Physical therapy- general classification
	0421	Physical therapy-visit charge
	0422	Physical therapy-hourly charge
	0423	Physical therapy-group rate
	0424	Physical therapy-evaluation or re-evaluation
	0429	Physical therapy-other
Diagnosis-Related Group (DRG) to identify lower extremity joint replacement procedure	469	Major hip and knee joint replacement ore reattachment of lower extremity with major complications or comorbidities (MCC)
	470	Major hip and knee joint replacement ore reattachment of lower extremity without MCC

TABLE 2 Association between social determinants of health and home health physical therapy visits for patients discharged to home using generalized linear model analysis with a gamma distribution.

	Hip Replacement		Knee Replacement	
	Estimated Coefficients (95% CI)	*P* value	Estimated Coefficients (95% CI)	*P* value
Race (ref: White)				
Black	0.51 (0.25, 0.76)	**<0.001**	0.35 (0.15, 0.55)	**0.001**
Other race	-0.10 (-0.35, 0.14)	0.382	0.42 (0.24, 0.59)	**<0.001**
Ref: Non-dual eligible				
Dual Eligible Status	1.28 (0.99, 1.57)	**<0.001**	0.74 (0.54, 0.93)	**<0.001**
Hospital DPP	0.61 (0.11, 1.11)	**0.017**	1.19 (0.79, 1.60)	**<0.001**
Social Vulnerability Index (SVI) Themes				
Socioeconomic Status	1.00 (0.75, 1.26)	**<0.001**	0.41 (0.19, 0.62)	**<0.001**
Household Characteristics	-0.05 (-0.21, 0.20)	0.964	0.11 (-0.07, 0.29)	0.221
Racial and Ethnicity	-0.50 (-0.80, -0.21)	**0.002**	-0.15 (-0.40, 0.08)	0.197
Housing type & Transportation	0.29 (0.08, 0.49)	**0.007**	0.79 (0.62, 0.97)	**<0.001**

P-values < 0.05 in bold
Abbreviations: DPP, Disproportionate Share Hospital patient percentage; SVI, social vulnerability index

TABLE 3 Association between social determinants of health and outpatient physical therapy visits for patients discharged to home using generalized linear model analysis with a gamma distribution.

	Hip Replacement		Knee Replacement	
	Estimated Coefficients (95% CI)	*P* value		Estimated Coefficients (95% CI)
Race (ref: White)				
Black	0.90 (0.20, 1.59)	**0.012**	0.59 (0.09, 1.11)	**0.022**
Other race	0.78 (0.04, 1.50)	**0.038**	0.36 (-0.12, 0.85)	0.142
Ref: Non-dual eligible				
Dual Eligible Status	-0.82 (-1.51, -0.12)	**0.022**	-2.12 (-2.52, -1.73)	**<0.001**
Hospital DPP	-3.24 (-4.69, -1.80)	**<0.001**	-3.11 (-4.17, -2.06)	**<0.001**
Social Vulnerability Index (SVI) Themes				
Socioeconomic Status	-0.15 (-0.88, 0.59)	0.704	0.84 (0.27, 1.41)	**0.004**
Household Characteristics	0.17 (-0.43, 0.77)	0.585	0.40 (-0.06, 0.85)	0.089
Racial & Ethnicity	0.42 (-0.44, 1.28)	0.339	-0.31 (-0.96, 0.33)	0.338
Housing type & Transportation	-0.69 (-1.28, -0.10)	**0.021**	-0.72 (-1.18, -0.27)	**0.002**

P-values < 0.05 in bold

Abbreviations: DPP, Disproportionate Share Hospital patient percentage; SVI, social vulnerability index

TABLE 4 (Supplement): Variance Inflation Factor for covariates included in the full regression models.

		Home health physical therapy visits		Outpatient physical therapy visits	
		Hip	Knee	Hip	Knee
	Variables	Variance Inflation Factor			
Patient level	CJR	1.15	1.14	1.14	1.10
	Age Group (ref: Age 66-69)				
	Age 70-74	1.40	1.41	1.41	1.41
	Age 75-79	1.37	1.38	1.37	1.38
	Age 80-84	1.25	1.24	1.25	1.24
	Age 85+	1.13	1.10	1.13	1.09
	Race (ref: White)				
	Black	1.03	1.04	1.05	1.05
	Other race	1.03	1.10	1.06	1.10
	Sex (ref: Male)				
	Female	1.02	1.02	1.03	1.02
	CCI (ref: CCI:0)				
	CCI 1	1.14	1.10	1.15	1.17
	CCI 2+	1.18	1.20	1.21	1.20
	MS DRG (ref: 469)				
	MS-DRG 470	1.01	1.01	1.02	1.01
	Dual eligibility (ref: non-dual eligible)	1.06	1.15	1.08	1.15
Hospital level	For-profit hospital (ref: profit)	1.37	1.53	1.41	1.63
	DPP	1.94	2.01	1.94	1.97
	Census as percent of capacity	1.92	1.95	1.89	1.86
	LEJR volume as % of census	2.36	2.66	2.20	2.54
	Teaching hospital (ref: teaching)	1.58	1.55	1.66	1.61

	Bed (ref: bed 100 or less)				
	Bed 101-200	2.73	2.55	2.76	2.51
	Bed 201-300	3.50	3.17	3.25	2.89
	Bed 301-400	3.25	2.90	3.06	2.85
	Bed 401-500	2.51	2.62	2.20	2.37
	Bed 501-600	2.07	2.05	2.08	2.02
	Bed 601-700	2.32	2.22	2.19	2.14
	Bed 701 or more	3.92	3.52	3.97	3.44
Regional level	Socioeconomic (SVI)	2.33	2.32	2.39	2.37
	Household Characteristics (SVI)	1.40	1.48	1.44	1.46
	Racial & Ethnic minority (SVI)	2.02	1.96	2.16	2.05
	Housing type & Transportation (SVI)	1.63	1.59	1.62	1.57

Abbreviations: CJR, Comprehensive Care for Joint Replacement; CCI, Charlson Comorbidity Index; MS-DRG, Medicare severity diagnosis related group; DPP, Disproportionate Share Hospital patient percentage; LEJR, lower extremity joint replacement; SVI, social vulnerability index

TABLE 5 (Supplement): Multivariate analyses of home health physical therapy visits with covariates related to social determinants of health for patients regardless of discharge destination.

	Hip		Knee	
	Estimated Coefficients (95% CI)	*P* value	Estimated Coefficients (95% CI)	*P* value
Race (ref: White)				
Black	0.50 (0.22, 0.73)	**<0.001**	0.56 (0.36, 0.76)	**<0.001**
Other race	0.02 (-0.21, 0.25)	0.866	0.42 (0.26, 0.61)	**<0.001**
Dual Eligible Status (ref: non-dual eligible)	1.23 (0.98, 1.49)	**<0.001**	0.67 (0.50, 0.86)	**<0.001**
Hospital DPP	0.86 (0.40, 1.32)	**<0.001**	1.30 (0.95, 1.65)	**<0.001**
Social Vulnerability Index (SVI) Themes				
Socioeconomic Status	0.01 (-0.02, 0.04)	0.600	0.01 (0.00, 0.01)	**0.036**
Household Characteristics	0.06 (-0.09, 0.21)	0.438	-0.16 (-0.28, -0.03)	**0.013**
Racial and Ethnic Minority Status	-0.01 (-0.00, 0.02)	0.945	0.01 (-0.01, 0.02)	0.424
Housing type and Transportation	-0.06 (0.21, 0.09)	0.430	0.16 (0.03, 0.28)	**0.014**

P-values < 0.05 in bold
Abbreviations: DPP, Disproportionate Share Hospital patient percentage; SVI, social vulnerability index

TABLE 6 (Supplement): Multivariate analyses of home health physical therapy visits with patient level covariates for patients discharged home.

	Home health physical therapy visits			
	Hip		Knee	
Variables	Estimated Coefficients (95% CI)	*P* value	Estimated Coefficients (95% CI)	*P* value
CJR	-0.65 (-0.76, -0.55)	**<0.001**	-0.82 (-0.90, -0.73)	**<0.001**
Age Group (ref: Age 66-69)				
Age 70-74	0.26 (0.13, 0.38)	**<0.001**	0.16 (0.05, 0.27)	**0.003**
Age 75-79	0.70 (0.56, 0.85)	**<0.001**	0.39 (0.27, 0.51)	**<0.001**
Age 80-84	1.19 (1.00, 1.39)	**<0.001**	0.82 (0.66, 0.98)	**<0.001**
Age 85+	1.91 (1.61, 2.20)	**<0.001**	0.97 (0.71, 1.22)	**<0.001**
Race (ref: White)				
Black	0.66 (0.38, 0.94)	**<0.001**	0.54 (0.31, 0.77)	**<0.001**
Other race	-0.09 (-0.34, 0.15)	0.449	0.37 (0.18, 0.56)	**<0.001**
Sex (ref: Male)				
Female	0.81 (0.70, 0.91)	**<0.001**	0.47 (0.38, 0.56)	**<0.001**
CCI (ref: CCI:0)				
CCI 1	0.49 (0.36, 0.63)	**<0.001**	0.15 (0.04, 0.26)	**0.007**
CCI 2+	0.68 (0.55, 0.81)	**<0.001**	0.37 (0.27, 0.48)	**<0.001**
MS DRG (ref: 469)				
MS-DRG 470	-0.94 (-1.49, -0.40)	**0.001**	0.35 (-0.03, 0.73)	0.071
Dual eligibility (ref: non-dual eligible)	1.34 (1.03, 1.66)	**<0.001**	0.89 (0.68, 1.10)	**<0.001**

P-values < 0.05 in bold
Abbreviations: CJR, Comprehensive Care for Joint Replacement; CCI, Charlson Comorbidity Index; MS-DRG, Medicare severity diagnosis related group

TABLE 7 (Supplement): Multivariate analyses of home health physical therapy visits with patient level covariates for patients regardless of discharge destination.

	Home health physical therapy visits			
	Hip		Knee	
Variables	Estimated Coefficients (95% CI)	*P* value	Estimated Coefficients (95% CI)	*P* value
CJR	-0.58 (-0.68, -0.48)	**<0.001**	-0.85 (-0.93, -0.76)	**<0.001**
Age Group (ref: Age 66-69)				
Age 70-74	0.28 (0.16, 0.40)	**<0.001**	0.14 (0.04, 0.24)	**0.007**
Age 75-79	0.83 (0.69, 0.97)	**<0.001**	0.44 (0.32, 0.55)	**<0.001**
Age 80-84	1.37 (1.19, 1.54)	**<0.001**	0.76 (0.62, 0.90)	**<0.001**
Age 85+	2.35 (2.12, 2.58)	**<0.001**	1.29 (1.07, 1.49)	**<0.001**
Race (ref: White)				
Black	0.61 (0.35, 0.86)	**<0.001**	0.67 (0.46, 0.87)	**<0.001**
Other race	0.13 (-0.12, 0.37)	0.313	0.39 (0.21, 0.57)	**<0.001**
Sex (ref: Male)				
Female	0.87 (0.77, 0.97)	**<0.001**	0.52 (0.43, 0.60)	**<0.001**
CCI (ref: CCI:0)				
CCI 1	0.50 (0.38, 0.63)	**<0.001**	0.20 (0.10, 0.30)	**<0.001**
CCI 2+	0.76 (0.64, 0.88)	**<0.001**	0.44 (0.34, 0.53)	**<0.001**
MS DRG (ref: 469)				
MS-DRG 470	-1.44 (-1.87, -1.02)	**<0.001**	-0.07 (-0.37, 0.23)	0.652
Medicare-Medicaid dual eligibility (ref: non-dual eligible)	1.07 (0.81, 1.33)	**<0.001**	0.77 (0.60, 0.94)	**<0.001**

P-values < 0.05 in bold
Abbreviations: CJR, Comprehensive Care for Joint Replacement; CCI, Charlson Comorbidity Index; MS-DRG, Medicare severity diagnosis related group

TABLE 8 (Supplement): Multivariate analyses of home health physical therapy visits with patient, hospital, and regional level characteristics for patients discharged home.

		Home health physical therapy visits			
		Hip		Knee	
	Variables	Estimated Coefficients (95% CI)	*P* value	Estimated Coefficients (95% CI)	*P* value
Patient level	CJR	-0.74 (-0.86, -0.63)	**<0.001**	-0.97 (-1.06, -0.87)	**<0.001**
	Age Group (ref: Age 66-69)				
	Age 70-74	0.25 (0.13, 0.37)	**<0.001**	0.14 (0.04, 0.25)	**0.007**
	Age 75-79	0.71 (0.57, 0.85)	**<0.001**	0.42 (0.30, 0.53)	**<0.001**
	Age 80-84	1.27 (1.07, 1.46)	**<0.001**	0.84 (0.69, 1.00)	**<0.001**
	Age 85+	1.89 (1.60, 2.18)	**<0.001**	1.06 (0.81, 1.31)	**<0.001**
	Race (ref: White)				
	Black	0.53 (0.25, 0.81)	**<0.001**	0.37 (0.14, 0.60)	**0.001**
	Other race	-0.09 (-0.32, 0.15)	0.471	0.42 (0.22, 0.61)	**<0.001**
	Sex (ref: Male)				
	Female	0.85 (0.74, 0.95)	**<0.001**	0.48 (0.39, 0.56)	**<0.001**
	CCI (ref: CCI:0)				
	CCI 1	0.39 (0.26, 0.52)	**<0.001**	0.08 (-0.02, 0.19)	0.128
	CCI 2+	0.63 (0.51, 0.75)	**<0.001**	0.31 (0.21, 0.41)	**<0.001**
	MS DRG (ref: 469)				
	MS-DRG 470	-1.13 (-1.69, -0.57)	**<0.001**	0.29 (-0.09, 0.66)	0.139
	Dual eligibility (ref: non-dual eligible)	1.29 (0.98, 1.61)	**<0.001**	0.76 (0.54, 0.97)	**<0.001**
Hospital level	For-profit hospital (ref: profit)	1.08 (0.91, 1.26)	**<0.001**	0.99 (0.85, 1.13)	**<0.001**
	DPP	0.62 (0.10, 1.13)	**0.019**	1.06 (0.64, 1.47)	**<0.001**
	Census as percent of capacity	1.31 (0.84, 1.78)	**<0.001**	1.16 (0.79, 1.53)	**<0.001**
	LEJR volume as % of census	-0.22 (-0.60, 0.17)	0.268	0.31 (-0.02, 0.63)	0.063
	Teaching hospital (ref: teaching)	-0.17 (-0.29, -0.04)	**0.010**	0.01 (-0.10, 0.11)	0.896

	Bed (ref: bed 100 or less)				
	Bed 101-200	-0.60 (-0.82, -0.38)	**<0.001**	0.01 (-0.16, 0.19)	0.869
	Bed 201-300	-0.38 (-0.62, -0.14)	**0.002**	0.05 (-0.14, 0.24)	0.587
	Bed 301-400	-0.54 (-0.80, -0.28)	**<0.001**	-0.18 (-0.39, 0.03)	0.096
	Bed 401-500	-0.14 (-0.43, 0.16)	0.355	0.49 (0.26, 0.72)	**<0.001**
	Bed 501-600	0.01 (-0.32, 0.33)	0.982	0.10 (-0.17, 0.36)	0.474
	Bed 601-700	-1.02 (-1.34, -0.67)	**<0.001**	-0.52 (-0.78, -0.27)	**<0.001**
	Bed 701 or more	-0.55 (-0.84, -0.25)	**<0.001**	-0.46 (-0.70, -0.23)	**<0.001**
Regional level	Socioeconomic (SVI)	0.97 (0.71, 1.23)	**<0.001**	0.40 (0.18, 0.61)	**<0.001**
	Household Characteristics (SVI)	-0.03 (-0.25, 0.19)	0.797	0.07 (-0.11, 0.25)	0.471
	Racial & Ethnic minority (SVI)	-0.36 (-0.66, -0.05)	**0.022**	-0.02 (-0.27, 0.23)	0.873
	Housing type & Transportation (SVI)	0.33 (0.12, 0.54)	**0.002**	0.84 (0.67, 1.02)	**<0.001**

P-values < 0.05 in bold

Abbreviations: CJR, Comprehensive Care for Joint Replacement; CCI, Charlson Comorbidity Index; MS-DRG, Medicare severity diagnosis related group; DPP, Disproportionate Share Hospital patient percentage; LEJR, lower extremity joint replacement; SVI, social vulnerability index

TABLE 9 (Supplement): Multivariate analyses of home health physical therapy visits with patient, hospital, and regional level characteristics for patients regardless of discharge destination

		Home health physical therapy visits			
		Hip		Knee	
	Variables	Estimated Coefficients (95% CI)	*P* value	Estimated Coefficients (95% CI)	*P* value
Patient level	CJR	-0.69 (-0.79, -0.56)	**<0.001**	-0.97 (-1.06, -0.89)	**<0.001**
	Age Group (ref: Age 66-69)				
	Age 70-74	0.30 (0.18, 0.42)	**<0.001**	0.15 (0.05, 0.25)	**0.004**
	Age 75-79	0.91 (0.78, 1.05)	**<0.001**	0.44 (0.33, 0.55)	**<0.001**
	Age 80-84	1.55 (1.38, 1.72)	**<0.001**	0.79 (0.64, 0.92)	**<0.001**
	Age 85+	2.35 (2.12, 2.58)	**<0.001**	1.31 (1.10, 1.52)	**<0.001**
	Race (ref: White)				
	Black	0.50 (0.22, 0.73)	**<0.001**	0.56 (0.36, 0.76)	**<0.001**
	Other race	0.02 (-0.21, 0.25)	0.866	0.42 (0.26, 0.61)	**<0.001**
	Sex (ref: Male)				
	Female	0.92 (0.82, 1.01)	**<0.001**	0.53 (0.44, 0.61)	**<0.001**
	CCI (ref: CCI:0)				
	CCI 1	0.44 (0.31, 0.56)	**<0.001**	0.16 (0.06, 0.26)	**0.002**
	CCI 2+	0.78 (0.66, 0.90)	**<0.001**	0.41 (0.32, 0.51)	**<0.001**
	MS DRG (ref: 469)				
	MS-DRG 470	-1.35 (-1.78, -0.92)	**<0.001**	0.04 (-0.26, 0.33)	0.792
	Medicare-Medicaid dual eligibility (ref: non-dual eligible)	1.23 (0.98, 1.49)	**<0.001**	0.67 (0.50, 0.86)	**<0.001**
Hospital level	For-profit hospital (ref: profit)	0.98 (0.82, 1.14)	**<0.001**	1.01 (0.88, 1.13)	**<0.001**
	DPP	0.86 (0.40, 1.32)	**<0.001**	1.30 (0.95, 1.65)	**<0.001**
	Census as percent of capacity	1.04 (0.61, 1.48)	**<0.001**	0.63 (0.29, 0.97)	**<0.001**

	LEJR volume as % of census	-0.23 (-0.57, 0.11)	0.179	0.55 (0.27, 0.84)	**<0.001**
	Teaching hospital (ref: teaching)	-0.07 (-0.19, 0.65)	0.238	0.08 (-0.02, 0.18)	0.099
	Bed (ref: bed 100 or less)				
	Bed 101-200	-0.53 (-0.73, -0.32)	**<0.001**	0.18 (0.14, 0.34)	**0.033**
	Bed 201-300	-0.28 (-0.50, -0.06)	**0.012**	0.22 (0.05, 0.39)	**0.014**
	Bed 301-400	-0.56 (-0.80, -0.31)	**<0.001**	0.05 (-0.14, 0.25)	0.602
	Bed 401-500	-0.14 (-0.41, 0.14)	0.317	0.62 (0.41, 0.83)	**<0.001**
	Bed 501-600	0.03 (-0.28, 0.34)	0.854	0.40 (0.16, 0.64)	**0.001**
	Bed 601-700	-0.78 (-1.09, -0.47)	**<0.001**	-0.10 (-0.34, 0.14)	0.408
	Bed 701 or more	-0.55 (-0.82, -0.27)	**<0.001**	-0.22 (-0.44, -0.00)	**0.046**
Regional level	Socioeconomic (SVI)	0.01 (-0.02, 0.04)	0.600	0.01 (0.00, 0.01)	**0.036**
	Household Characteristics (SVI)	0.06 (-0.09, 0.21)	0.438	-0.16 (-0.28, -0.03)	**0.013**
	Racial & Ethnic minority (SVI)	-0.01 (-0.00, 0.02)	0.945	0.01 (-0.01, 0.02)	0.424
	Housing type & Transportation (SVI)	-0.06 (0.21, 0.09)	0.430	0.16 (0.03, 0.28)	**0.014**

P-values < 0.05 in bold

Abbreviations: CJR, Comprehensive Care for Joint Replacement; CCI, Charlson Comorbidity Index; MS-DRG, Medicare severity diagnosis related group; DPP, Disproportionate Share Hospital patient percentage; LEJR, lower extremity joint replacement; SVI, social vulnerability index

TABLE 10 (Supplement) Multivariate analyses of outpatient physical therapy visits with covariates related to social determinants of health for patients regardless of discharge destination.

	Hip		Knee	
	Estimated Coefficients (95% CI)	*P* value	Estimated Coefficients (95% CI)	*P* value
Race (ref: White)				
Black	0.87 (0.22, 1.53)	**0.009**	-0.08 (-0.52, 0.35)	0.706
Other race	0.70 (0.03, 1.37)	**0.041**	0.28 (-0.15, 0.72)	0.201
Dual Eligible Status (ref: non-dual eligible)	-1.39 (-2.03, -0.74)	**<0.001**	-2.15 (-2.54, -1.76)	**<0.001**
Hospital DPP	-3.57 (-4.82, -2.34)	**<0.001**	-3.04 (-3.88, -2.20)	**<0.001**
Social Vulnerability Index (SVI) Themes				
Socioeconomic Status	0.01 (-0.00, 0.01)	**<0.001**	-0.01 (-0.01, 0.01)	0.092
Household Characteristics	0.43 (0.03, 0.84)	**0.036**	0.32 (0.01, 0.62)	**0.044**
Racial & Ethnic Minority Status	0.02 (-0.06, 0.05)	0.145	0.01 (-0.03, 0.03)	0.925
Housing type & Transportation	-0.44 (-0.85, -0.04)	**0.033**	-0.31 (-0.62, -0.01)	**0.046**

P-values < 0.05 in bold
Abbreviations: DPP, Disproportionate Share Hospital patient percentage; SVI, social vulnerability index

TABLE 11 (Supplement): Multivariate analyses of outpatient physical therapy visits with patient level covariates for patients discharged home.

	Outpatient physical therapy visits			
	Hip		Knee	
Variables	Estimated Coefficients (95% CI)	*P* value	Estimated Coefficients (95% CI)	*P* value
CJR	0.38 (0.09, 0.67)	**0.010**	0.58 (0.36, 0.80)	**<0.001**
Age Group (ref: Age 66-69)				
Age 70-74	-0.23 (-0.60, 0.13)	0.210	-0.03 (-0.31, 0.25)	0.857
Age 75-79	-0.16 (-0.57, 0.24)	0.432	-0.60 (-0.90, -0.30)	**<0.001**
Age 80-84	-0.54 (-1.05, -0.03)	**0.038**	-0.82 (-1.20, -0.44)	**<0.001**
Age 85+	-0.71 (-1.46, 0.03)	0.061	-1.36 (-1.93, -0.78)	**<0.001**
Race (ref: White)				
Black	1.10 (0.39, 1.82)	**0.003**	0.78 (0.26, 1.30)	**0.003**
Other race	0.38 (-0.35, 1.12)	0.308	0.30 (-0.19, 0.78)	0.228
Sex (ref: Male)				
Female	-0.13 (-0.43, 0.16)	0.375	-0.22 (-0.44, 0.01)	0.062
CCI (ref: CCI:0)				
CCI 1	-0.19 (-0.57, 0.18)	0.315	0.01 (-0.27, 0.28)	0.979
CCI 2+	-0.04 (-0.39, 0.30)	0.811	-0.18 (-0.44, -0.08)	0.169
MS DRG (ref: 469)				
MS-DRG 470	1.73 (0.39, 3.07)	**0.012**	1.29 (0.49, 2.09)	**0.002**
Dual eligibility (ref: non-dual eligible)	-1.37 (-2.14, -0.59)	**0.001**	-2.81 (-3.25, -2.36)	**<0.001**

P-values < 0.05 in bold

Abbreviations: CJR, Comprehensive Care for Joint Replacement; CCI, Charlson Comorbidity Index; MS-DRG, Medicare severity diagnosis related group

TABLE 12 (Supplement): Multivariate analyses of outpatient physical therapy visits with patient level covariates for patients regardless of discharge destination.

	Outpatient physical therapy visits			
	Hip		Knee	
Variables	Estimated Coefficients (95% CI)	*P* value	Estimated Coefficients (95% CI)	*P* value
CJR	0.39 (0.13, 0.66)	**0.003**	0.61 (0.42, 0.81)	**<0.001**
Age Group (ref: Age 66-69)				
Age 70-74	-0.21 (-0.55, 0.14)	0.237	-0.15 (-0.40, 0.11)	0.267
Age 75-79	-0.35 (-0.73, 0.02)	0.065	-0.73 (-1.00, -0.46)	**<0.001**
Age 80-84	-0.76 (-1.20, -0.32)	**0.001**	-1.20 (-1.53, -0.88)	**<0.001**
Age 85+	-1.06 (-1.64, -0.48)	**<0.001**	-2.23 (-2.69, -1.78)	**<0.001**
Race (ref: White)				
Black	1.00 (0.33, 1.67)	**0.003**	0.11 (-0.32, 0.54)	0.629
Other race	0.34 (-0.36, 1.03)	0.341	0.22 (-0.22, 0.66)	0.321
Sex (ref: Male)				
Female	-0.25 (-0.52, 0.02)	0.072	-0.50 (-0.71, -0.29)	**<0.001**
CCI (ref: CCI:0)				
CCI 1	-0.14 (-0.48, 0.20)	0.412	-0.15 (-0.41, 0.10)	0.228
CCI 2+	-0.24 (-0.55, 0.07)	0.127	-0.49 (-0.71, -0.26)	**<0.001**
MS DRG (ref: 469)				
MS-DRG 470	2.12 (1.13, 3.11)	**<0.001**	1.36 (0.74, 1.99)	**<0.001**
Medicare-Medicaid dual eligibility (ref: non-dual eligible)	-1.71 (-2.38, -1.05)	**<0.001**	-2.83 (-3.21, -2.46)	**<0.001**

P-values < 0.05 in bold

Abbreviations: CJR, Comprehensive Care for Joint Replacement; CCI, Charlson Comorbidity Index; MS-DRG, Medicare severity diagnosis related group

TABLE 13 (Supplement): Multivariate analyses of outpatient physical therapy visits with patient, hospital, and regional level characteristics for patients discharged home

		Outpatient physical therapy visits			
		Hip		Knee	
	Variables	Estimated Coefficients (95% CI)	*P* value	Estimated Coefficients (95% CI)	*P* value
Patient level	CJR	0.15 (-0.16, 0.45)	0.341	0.36 (0.13, 0.58)	**0.002**
	Age Group (ref: Age 66-69)				
	Age 70-74	-0.16 (-0.52, 0.20)	0.384	0.06 (-0.22, 0.34)	0.692
	Age 75-79	-0.07 (-0.47, 0.33)	0.743	-0.56 (-0.86, -0.26)	**<0.001**
	Age 80-84	-0.42 (-0.93, 0.08)	0.099	-0.83 (-1.21, -0.46)	**<0.001**
	Age 85+	-0.72 (-1.45, 0.01)	0.053	-1.32 (-1.89, -0.75)	**<0.001**
	Race (ref: White)				
	Black	0.92 (0.20, 1.64)	**0.012**	0.56 (0.03, 1.09)	**0.040**
	Other race	0.78 (0.07, 1.50)	**0.032**	0.33 (-0.16, 0.81)	0.189
	Sex (ref: Male)				
	Female	0.03 (-0.27, 0.33)	0.843	-0.16 (-0.39, 0.06)	0.158
	CCI (ref: CCI:0)				
	CCI 1	-0.32 (-0.69, 0.05)	0.091	-0.07 (-0.34, 0.21)	0.624
	CCI 2+	-0.18 (-0.51, 0.16)	0.310	-0.13 (-0.38, 0.13)	0.330
	MS DRG (ref: 469)				
	MS-DRG 470	1.24 (-0.05, 2.52)	0.059	1.22 (0.43, 2.01)	**0.003**
	Dual eligibility (ref: non-dual eligible)	-0.86 (-1.63, -0.09)	**0.028**	-2.04 (-2.50, -1.57)	**<0.001**
Hospital level	For-profit hospital (ref: profit)	0.47 (0.01, 0.93)	**0.047**	0.68 (0.32, 1.03)	**<0.001**
	DPP	-3.14 (-4.67, -1.61)	**<0.001**	-2.94 (-3.99, -1.88)	**<0.001**
	Census as percent of capacity	0.82 (-0.43, 2.06)	0.198	0.68 (-0.23, 1.59)	0.145
	LEJR volume as % of census	-2.00 (-3.03, -0.96)	**<0.001**	-0.27 (-1.06, 0.52)	0.497

	Teaching hospital (ref: teaching)	-0.09 (-0.45, 0.27)	0.619	-0.20 (-0.47, 0.07)	0.138
	Bed (ref: bed 100 or less)				
	Bed 101-200	-0.21 (-0.77, 0.35)	0.469	0.13 (-0.29, 0.55)	0.537
	Bed 201-300	-0.41 (-1.05, 0.22)	0.200	0.42 (-0.06, 0.90)	0.084
	Bed 301-400	-0.25 (-0.94, 0.44)	0.482	1.01 (0.51, 1.51)	**<0.001**
	Bed 401-500	-0.54 (-1.35, 0.28)	0.196	-0.12 (-0.69, 0.44)	0.668
	Bed 501-600	-0.58 (-1.42, 0.25)	0.171	0.78 (0.15, 1.42)	**0.016**
	Bed 601-700	-0.85 (-1.71, 0.02)	**0.055**	0.28 (-0.37, 0.92)	0.404
	Bed 701 or more	0.19 (-0.58, 0.96)	0.621	1.41 (0.83, 1.98)	**<0.001**
Regional level	Socioeconomic (SVI)	-0.20 (-0.96, 0.56)	0.603	0.72 (0.14, 1.30)	**0.014**
	Household Characteristics (SVI)	0.26 (-0.33, 0.86)	0.380	0.41 (-0.04, 0.85)	0.076
	Racial & Ethnic minority (SVI)	0.36 (-0.52, 1.23)	0.425	-0.24 (-0.89, 0.41)	0.470
	Housing type & Transportation (SVI)	-0.70 (-1.29, -0.11)	**0.021**	-0.66 (-1.11, -0.20)	**0.005**

P-values < 0.05 in bold

Abbreviations: CJR, Comprehensive Care for Joint Replacement; CCI, Charlson Comorbidity Index; MS-DRG, Medicare severity diagnosis related group; DPP, Disproportionate Share Hospital patient percentage; LEJR, lower extremity joint replacement; SVI, social vulnerability index

TABLE 14 (Supplement): Multivariate analyses of outpatient physical therapy visits with patient, hospital, and regional level characteristics for patients regardless of discharge destination.

		Outpatient physical therapy visits			
		Hip		Knee	
	Variables	Estimated Coefficients (95% CI)	*P* value	Estimated Coefficients (95% CI)	*P* value
Patient level	CJR	0.18 (-0.09, 0.44)	0.191	0.44 (0.24, 0.64)	**<0.001**
	Age Group (ref: Age 66-69)				
	Age 70-74	-0.19 (-0.52, 0.14)	0.267	-0.07 (-0.33, 0.19)	0.611
	Age 75-79	-0.29 (-0.66, 0.08)	0.123	-0.66 (-0.93, -0.39)	**<0.001**
	Age 80-84	-0.80 (-1.23, -0.37)	**<0.001**	-1.28 (-1.60, -0.96)	**<0.001**
	Age 85+	-1.14 (-1.70, -0.58)	**<0.001**	-2.23 (-2.68, -1.79)	**<0.001**
	Race (ref: White)				
	Black	0.87 (0.22, 1.53)	**0.009**	-0.08 (-0.52, 0.35)	0.706
	Other race	0.70 (0.03, 1.37)	**0.041**	0.28 (-0.15, 0.72)	0.201
	Sex (ref: Male)				
	Female	-0.06 (-0.32, 0.21)	0.674	-0.45 (-0.65, -0.24)	**<0.001**
	CCI (ref: CCI:0)				
	CCI 1	-0.21 (-0.54, 0.12)	0.217	-0.17 (-0.42, 0.88)	0.189
	CCI 2+	-0.32 (-0.61, -0.18)	**0.038**	-0.45 (-0.67, -0.23)	**<0.001**
	MS DRG (ref: 469)				
	MS-DRG 470	1.73 (0.76, 2.70)	**<0.001**	1.30 (0.67, 1.94)	**<0.001**
	Medicare-Medicaid dual eligibility (ref: non-dual eligible)	-1.39 (-2.03, -0.74)	**<0.001**	-2.15 (-2.54, -1.76)	**<0.001**

Hospital level	For-profit hospital (ref: profit)	0.03 (-0.37, 0.44)	0.885	0.46 (0.15, 0.77)	**0.003**
	DPP	-3.57 (-4.82, -2.34)	**<0.001**	-3.04 (-3.88, -2.20)	**<0.001**
	Census as percent of capacity	0.56 (-0.56, 1.67)	0.331	0.49 (-0.31, 1.29)	0.233
	LEJR volume as % of census	-1.88 (-2.75, -1.01)	**<0.001**	-0.29 (-0.97, 0.38)	0.393
	Teaching hospital (ref: teaching)	-0.13 (-0.44, 0.19)	0.431	-0.30 (-0.54, -0.07)	**0.012**
	Bed (ref: bed 100 or less)				
	Bed 101-200	-0.30 (-0.80, 0.20)	0.235	0.07 (-0.29, 0.44)	0.689
	Bed 201-300	-0.52 (-1.04, 0.10)	0.073	0.19 (-0.23, 0.60)	0.379
	Bed 301-400	-0.21 (-0.82, 0.41)	0.510	0.75 (0.31, 1.19)	**0.001**
	Bed 401-500	-0.49 (-1.21, 0.23)	0.178	-0.16 (-0.65, 0.35)	0.542
	Bed 501-600	-0.98 (-1.73, -0.23)	**0.010**	0.48 (-0.07, 1.04)	0.089
	Bed 601-700	-0.97 (-1.74, -0.20)	**0.013**	0.43 (-0.16, 1.01)	0.157
	Bed 701 or more	0.16 (-0.52, 0.84)	0.642	1.22 (0.72, 1.72)	**<0.001**
Regional level	Socioeconomic (SVI)	0.01 (-0.00, 0.01)	**<0.001**	-0.01 (-0.01, 0.01)	0.092
	Household Characteristics (SVI)	0.43 (0.03, 0.84)	**0.036**	0.32 (0.01, 0.62)	**0.044**
	Racial & Ethnic minority (SVI)	0.02 (-0.06, 0.05)	0.145	0.01 (-0.03, 0.03)	0.925
	Housing type & Transportation (SVI)	-0.44 (-0.85, -0.04)	**0.033**	-0.31 (-0.62, -0.01)	**0.046**

P-values < 0.05 in bold
Abbreviations: CJR, Comprehensive Care for Joint Replacement; CCI, Charlson Comorbidity Index; MS-DRG, Medicare severity diagnosis related group; DPP, Disproportionate Share Hospital patient percentage; LEJR, lower extremity joint replacement; SVI, social vulnerability index

FIGURE (Supplement). Exclusion criteria flow diagram

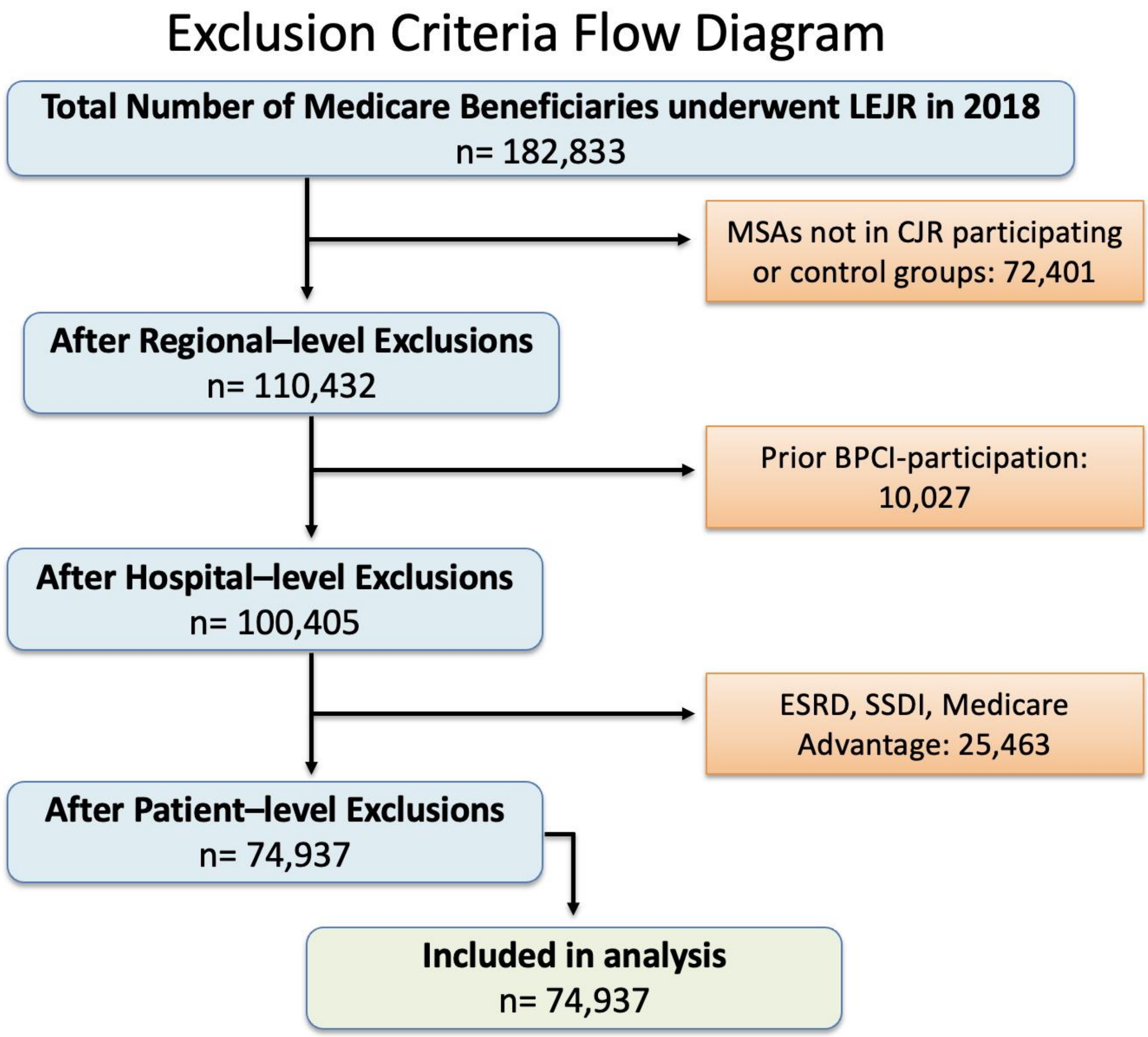

Supplement: Supplementary file 1 [file ptj-2025-0855_r1_supplementalfiles_sdohinpt_editorialofficeversion_pzag090.pdf]
